# Congruent geographic variation in saccular otolith shape across multiple species of African cichlids

**DOI:** 10.1038/s41598-020-69701-9

**Published:** 2020-07-30

**Authors:** Aneesh P. H. Bose, Holger Zimmermann, Georg Winkler, Alexandra Kaufmann, Thomas Strohmeier, Stephan Koblmüller, Kristina M. Sefc

**Affiliations:** 1 Institute of Biology, University of Graz, Universitätsplatz 2, 8010 Graz, Austria; 2 Department of Collective Behaviour, Max Planck Institute of Animal Behavior, Konstanz, Germany; 3 Centre for the Advanced Study of Collective Behaviour, University of Konstanz, Konstanz, Germany; 4 Department of Biology, University of Konstanz, Konstanz, Germany

**Keywords:** Ichthyology, Population genetics, Evolutionary ecology

## Abstract

The otoliths of teleost fishes exhibit a great deal of inter- and intra-species shape variation. The ecomorphology of the saccular otolith is often studied by comparing its shape across species and populations inhabiting a range of environments. However, formal tests are often lacking to examine how closely variation in otolith shape follows the genetic drift of a neutral trait. Here, we examine patterns of saccular otolith shape variation in four species of African cichlid fishes, each sampled from three field sites. All four species showed the greatest level of otolith shape variation along two principal component axes, one pertaining to otolith height and another to the prominence of an anterior notch. Fish collected from the same site possessed similarities in saccular otolith shape relative to fish from other sites, and these ‘site-difference’ signatures were consistent across species and observable in both sexes. Sex-differences in saccular otolith shape differed in magnitude from site to site. Population differences in saccular otolith shape did not covary with neutral genetic differentiation between those populations. Otolith height, in particular, displayed large site similarities across species, weak correlation with neutral genetic variation, and strong sex differences, collectively suggesting that otolith shape represents a selectively non-neutral trait.

## Introduction

The otoliths of the inner ears of teleost fishes represent a powerful lens for studying many aspects of fish biology including their ecology, neurobiology, bioacoustics, systematics, and fisheries stock assessments^1–3^. The morphology of teleost otoliths, i.e. their size and shape, is of particular interest, especially with respect to the saccular otolith. This is because the saccular otolith often displays high levels of morphological differentiation between species, and also notable differentiation within species, making it an important tool for fish biologists^4^. Saccular otolith shape alone can aid in discriminating between fish species (e.g. *Serranus* spp.^5^; rockfish spp.^6^), and also between stocks/populations, sexes, age-classes, and reproductive morphs (e.g. Atlantic cod, *Gadus morhua*^7^; plainfin midshipman fish, *Porichthys notatus*^8^; flatfish spp.^9^; round goby, *Neogobius melanostomus*^10^). Yet, the processes that give rise to such specific otolith shapes remain largely unknown, and represent a major outstanding evolutionary question about the structure and function of teleost otoliths^3^.

Intraspecific shape variation in saccular otoliths can be affected by a multitude of factors, including developmental, genetic, and environmental factors. While the saccular otoliths of young fish are often relatively simple in form, they frequently take on more complex, species-specific shapes as the fish grow^4,11^. Otolith shape is also, in part, genetically determined, as exemplified by Vignon and Morat^12^, who showed that coral reef snapper, *Lutjanus kasmira*, that live and grow under the same environmental conditions can have differently shaped saccular otoliths based on their genetic lineage (i.e. which population their ancestors historically belonged to). Within-species otolith shape can also be affected by diet (reef fish spp., *Amphiprion akindynos* and *Pomacentrus amboinensis*^13^) and rearing temperature (e.g. red drum, *Sciaenops ocellatus*^14^), illustrating the potential for local biotic and abiotic conditions to affect otolith shape as well.

The complexity of saccular otoliths and their interspecific morphological differences insinuate a biological meaning for otolith shape^1,3,15^. Otolith shape, in combination with other variables (including otolith mass, sulcus acusticus dimensions, and endolymph viscosity^3^), is expected to influence how otoliths oscillate within the end organs of the inner ear, thereby potentially impacting hearing capabilities^16–18^. Otolith shape, particularly of the saccular otolith, may therefore be one of many traits within the fish auditory system that collectively tune the hearing capabilities of fish to better suit their acoustic environment through local adaptation^1,3^. When viewing the immense variation, and intra-species consistency, in saccular otolith shape, it has often been assumed that otolith shape is adaptive, moulded by selection and/or by environmentally-induced plasticity, to match the demands of a particular habitat^1,3^. It may therefore be expected that different species that overlap in the same habitat, and use similar bandwidths in the acoustic environment, will show similarities in their saccular otolith shapes due to the common soundscape. By analogous reasoning, populations within a species that experience different environments (soundscapes) would be predicted to diverge in otolith shape. These expectations hold for phenotypically plastic variation in otolith shape and also for genetically determined, and hence heritable and selectable, variation. Alternatively, variation in otolith shape may sometimes *not* reflect any adaptive significance; that is, shape could be selectively neutral with respect to the environment and the functioning of the ear^3^. If selectively neutral, but still genetically determined, differences in otolith shape between species and populations would be expected to covary with divergence times, rates of gene flow, and population sizes; in other words, be driven by random drift. Alternatively, if otolith shape were selectively neutral, and not genetically determined, then variation in otolith shape would be expected to be random with respect to both soundscape and population structure.

Here, we examine patterns of saccular otolith shape variation in relation to field site differences and population structure using several species of African cichlid fishes from Lake Tanganyika, East Africa. Lake Tanganyika cichlids offer a unique opportunity to study otolith morphology for two main reasons. Many species are comprised of multiple distinct populations that live in close geographic proximity to one another, but are reproductively isolated due to natural barriers. Such natural barriers can include stretches of open sand or deep-water basins, which pose obstacles for small-bodied demersal species that are naturally found in rocky littoral habitats where they establish territories and find shelter among crevasses. Degrees of isolation vary among species as some are more stenotopic and more affected by habitat discontinuities than others^19–22^. Furthermore, lake water level fluctuations over geological and evolutionary time scales have periodically altered the structure of the shoreline and displaced coastal populations leading to historical alternations between geographic isolation and secondary contact^23^. As a consequence, populations of littoral cichlids in Lake Tanganyika vary in their times since divergence from one another according on how they were affected by lake level fluctuations^24^. Intra- and interspecific variation in rates of gene flow and population divergence times among the cichlids of Lake Tanganyika therefore present researchers with an excellent opportunity to compare natural replicate populations that vary along a genetic differentiation gradient.

In this study, we investigated saccular otolith shape variation in four species of cichlids, *Neolamprologus caudopunctatus, Neolamprologus pulcher*, *Neolamprologus savoryi*, and *Variabilichromis moorii*, from three distinct field sites in the southern tip of Lake Tanganyika. In particular, we tested the ecomorphology hypothesis for saccular otolith shape. That is, we examined whether otolith shape exhibits patterns consistent with it being attuned to environmental variation. We first asked whether our study species showed saccular otolith shape similarities based on which field site they were sampled from. That is, we asked “is there a group-level signature in otolith shape that differs across field sites?” Next, we asked whether the differences that we observe in saccular otolith shape between pairs of populations can be explained by the degree of neutral genetic differentiation between those populations. If otolith shape varies in response to environmental conditions, i.e. is selectively non-neutral, we expected to find that species share similar shape differences across sites, and that differences in otolith shape between populations would be unrelated to neutral genetic differentiation. To this end, we analyze a large sample of saccular otoliths, and control carefully for ontogenetic changes, and sex-differences, in shape.

## Methods

### Selection of field sites and study species

**Figure 1:**
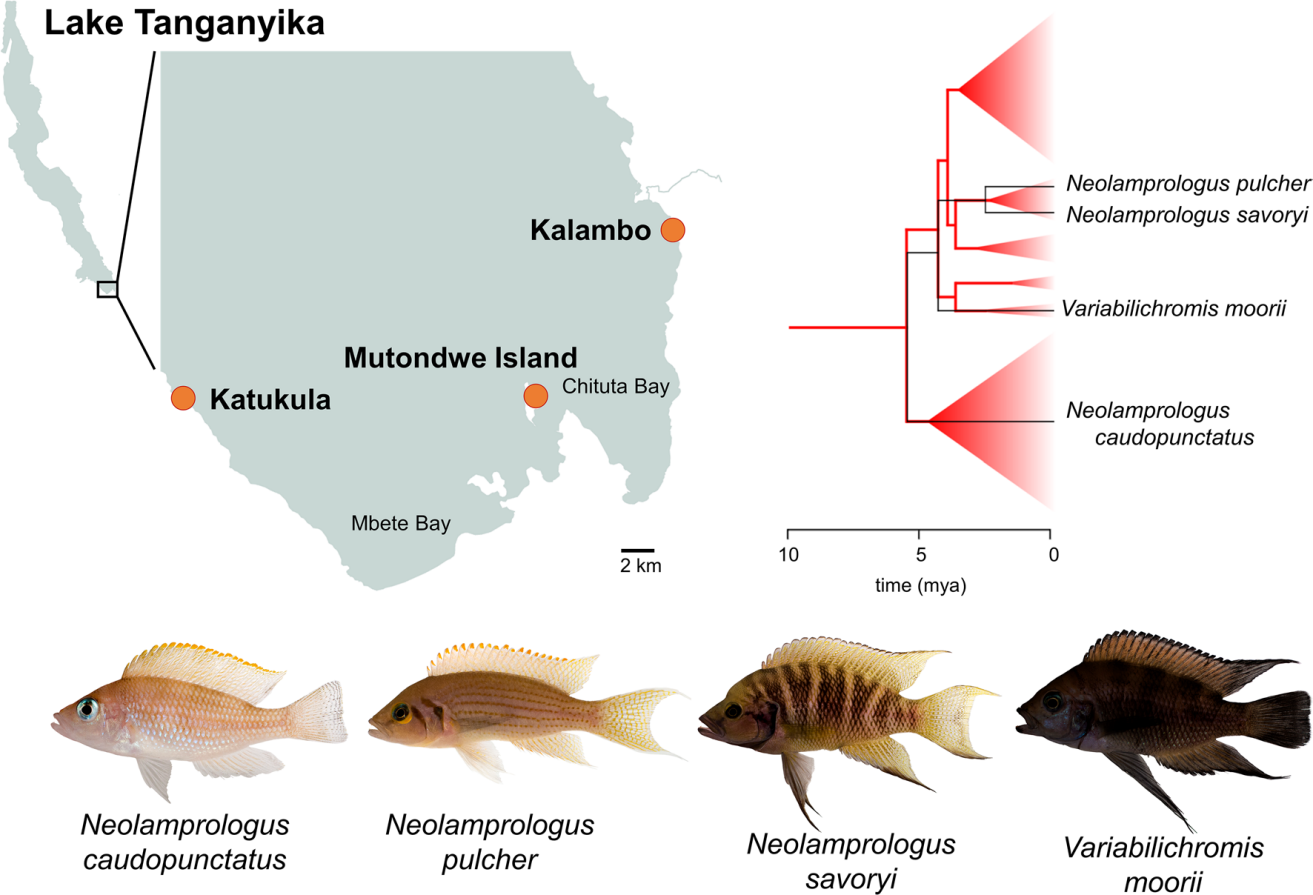
Field sites and study species. Top left: Map of the southern tip of Lake Tanganyika indicating the locations of the three field sites (orange dots). Top right: A schematic of the relationships and divergence times among the four study species (black lines) against a representation of the phylogenetic clades in the cichlid tribe Lamprogini (red). Triangle sizes represent the number of species within each phylogenetic clade. The divergence time estimates depicted in the diagram are from Irissari et al.^78^. Bottom: Photographs of the four study species (photo credit: Wolfgang Gessl, https://www.pisces.at).

We selected three field sites along the southern tip of Lake Tanganyika, Zambia (Fig. 1): Mutondwe Island (8° 42′ 29.4″ S 31° 07′ 18.0″ E), Kalambo Falls Lodge (8° 37′ 21.0″ S 31° 11′ 59.0″ E), and Katukula (8° 42′ 10.0″ S 30° 55′ 22.8″ E). All three sites are shallow-water, littoral, rocky habitats interspersed with sandy substrata. The sites do, however, differ with respect to the angle of the sloping shoreward incline (incline is shallow at Mutondwe Island, moderately steep at Katukula, and descends in steps at Kalambo), and the assortment of rocks (small rocks predominate at Mutondwe Island and Katukula, but rock size is larger and more variable at Kalambo). The Mutondwe Island site also represents a slightly more sheltered location relative to the more exposed shorelines of Katukula and Kalambo. The diversity and community of cichlid fishes sustained at each of these sites also varies subtly between the sites^25,26^. Our three field sites are separated from one another by heterogeneously structured habitat (e.g. the wide, sandy Mbete and Chituta Bays as well as the open water between the island and the shore sites). These natural barriers reduce gene flow and impose (some) reproductive isolation between the populations of demersal, rock-dwelling fish living there. In particular, previous population genetic and phylogeographic studies revealed that Mbete Bay persisted as a migration barrier for various demersal cichlid species throughout late-glacial lake level fluctuations^27^ and induced distinct genetic differentiation (in our study species^19,20,28^ and in other species^21,29,30^). In contrast, the bathymetric profile of the Chituta Bay area suggests that this bay merged into a continuous steeply inclining shore when the lake level dropped, which would make it permeable to connectivity among rock-dwelling cichlid populations. Indeed, genetic divergence across Chituta Bay is generally less pronounced^24,31^. Therefore, we expected the sampled populations to represent two levels of genetic differentiation within each species (stronger across Mbete Bay than across Chituta Bay). Additionally, levels of intraspecific population differentiation were expected to vary among species contingent on species-specific sensitivity to barriers and degrees of philopatry.

We selected four species to compare across the field sites: *N. caudopunctatus, N. pulcher*, *N. savoryi*, and *V. moorii*. These species were selected because they were particularly abundant at all locations and represent a sample of species that shares many similarities in ecology and niche-space. For example, all four species are small-bodied cichlids (typical standard lengths for breeding individuals are: 6.0 cm for *N. pulcher*^32^; 5.6 cm for *N. savoryi*^33^; 6.4 cm for *V. moorii*^34^; and 4.5–6 cm N*. caudopunctatus*^35^). Their distributions are all geographically limited to rocky littoral zones, and their depth ranges overlap (*N. pulcher*: 4–20 m, *N. savoryi*: 4–20 m, *N. caudopunctatus*: shallow—25 m^36^; *V. moorii*: 2–13 m^34,37^). Their diets are also similar, consisting of pelagic zooplankton, though *V. moorii* also commonly grazes on algae^36,37^. None of the species are piscivorous ambush or pursuit predators. All species are substrate breeders and prefer to live in groups that guard rocky territories covering relatively small areas (territories typically span < 4 m^2^^32,36,38^). Group sizes do, however, vary between the species as *V. moorii* is socially monogamous (group size is two), while average group size for *N. pulcher* ranges between seven^32^ and nine^39^. In *N. savoryi*, average group size is 14^33^. *N. caudopunctatus* also breed in pairs, but is considered colonial because they live at high densities^35^. *N. pulcher* has been documented to produce broadband high-frequency sounds (average ~ 12 kHz^40^), but sound production has not been investigated in any of our other species. None of our species have any documented swim-bladder specializations (e.g. the otophysan Weberian ossicles) linking air-filled compartments to the inner ear.

### Collection of specimens

**Figure 2:**
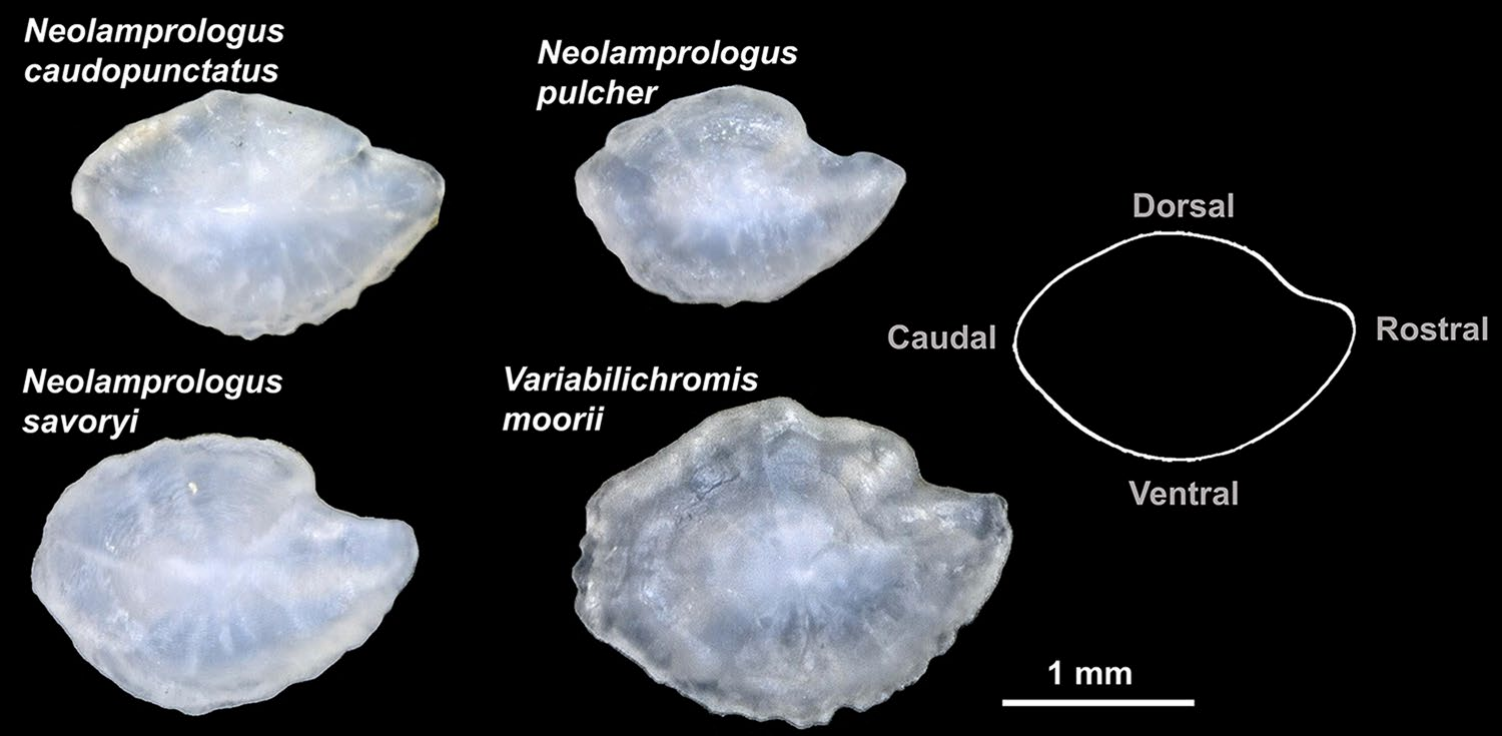
Examples of saccular otoliths from the four cichlid species investigated. The otolith surface in contact with the auditory sensory epithelium, i.e. the sulcus acusticus, is facing down. Outline on the right represents an average otolith contour and depicts the orientation of the otoliths within the fish. Note that the otoliths shown here are meant to represent variation across species not across field sites.

Using SCUBA and snorkeling, we sampled the same four species from each of our three field sites over two field seasons, between April 10–27 and September 19–October 6, 2018. In total, we collected 603 fish (see Table 1 for sample size broken down by field site and species). The fish were caught by hand using gill nets and were non-randomly selected by divers to cover a wide range of body sizes from both sexes. The fish were brought back to Mpulungu, Zambia and either dissected immediately or housed in outdoor holding tanks (~ 350 L) to be dissected within 10 days of initial capture. The fish were euthanized with an overdose of MS-222 (1 g/1 L lake water) and their sexes verified during dissections. Caudal fin clips were taken from each individual and stored in 99.9% ethanol. The two saccular otoliths from each fish were extracted, wiped clean, and stored dry (Fig. 2). Any otoliths that were vateritic (i.e. had distinctly crenulated margins) or were chipped during the handling process were not used in this study.

**Table 1:** Number of saccular otoliths used in this study split up by species, field site, and sex (female = F, male = M).

	Species				Total
	*Neolamprologus caudopunctatus*	*Neolamprologus pulcher*	*Neolamprologus savoryi*	*Variabilichromis moorii*	
**Field site**					
Mutondwe Island	N_F_ = 41 (24) N_M_ = 47 (25)	N_F_ = 49 (26) N_M_ = 41 (21)	N_F_ = 40 (24) N_M_ = 47 (29)	N_F_ = 33 (19) N_M_ = 91 (47)	N_F_ = 163 (93) N_M_ = 226 (122)
Kalambo	N_F_ = 40 (20) N_M_ = 53 (29)	N_F_ = 52 (28) N_M_ = 54 (28)	N_F_ = 43 (22) N_M_ = 39 (22)	N_F_ = 43 (26) N_M_ = 46 (24)	N_F_ = 178 (96) N_M_ = 192 (103)
Katukula	N_F_ = 40 (23) N_M_ = 45 (23)	N_F_ = 39 (20) N_M_ = 43 (25)	N_F_ = 38 (19) N_M_ = 64 (38)	N_F_ = 27 (18) N_M_ = 41 (23)	N_F_ = 144 (80) N_M_ = 193 (109)
Total	N_F_ = 121 (67) N_M_ = 145 (77)	N_F_ = 140 (74) N_M_ = 138 (74)	N_F_ = 121 (65) N_M_ = 150 (89)	N_F_ = 103 (63) N_M_ = 178 (94)	N = 1,096 (603)

The number of individuals contributing those otoliths is given in parentheses.

### Ethical note

All four species are highly abundant at our field sites and are not considered endangered (IUCN Red List, least concern for *N. pulcher*^41^, *N. caudopunctatus*^42^, *N. savoryi*^43^, and *V. moorii* [listed as *N. moorii*]^44^). This work was carried out in accordance with relevant guidelines and regulations under a study permit issued by the government of Zambia with permission from the Fisheries Department of Zambia and with approval from the ethics committee of the University of Graz (permit number 39/50/63 ex 2018/19). Fish euthanized in this study were also used in numerous additional research projects (e.g. Bose et al.^34^) including studies of parental care, mating behaviours, and parasite load.

### Saccular otolith shape quantification

Saccular otoliths from *N. caudopunctatus*, *N. pulcher*, *N. savoryi*, and *V. moorii* were all digitally photographed using a Keyence VHX-5000 digital microscope at 30× magnification (Fig. 2). Each otolith was placed with its sulcus acusticus facing downwards. As pairs of otoliths are approximate mirror images of one another, the photographs of each right saccular otolith were flipped horizontally, such that all images were of otoliths in the ‘left’ configuration. Otolith surface area was measured using the VHX Menu software associated with the Keyence VHX digital microscope.

For each species separately, we ran a Fourier power spectrum analysis to determine how many harmonics would be necessary to explain at least 99.999% of the variation in each species’ average otolith shape. *N. caudopunctatus* required 12 harmonics, *N. pulcher* and *N. savoryi* both required 11 harmonics, and *V. moorii* required 10 harmonics. We then ran a separate elliptic Fourier shape analysis for each species using the shape analysis software package SHAPE 1.3^45^. These analyses were followed with a principal component analysis (PCA) based on a variance–covariance matrix to reduce the shape variation within each species into a small number of principal components (PCs) that would each describe a different aspect of otolith shape (for more details on methodology and the use of elliptic Fourier shape analyses, see^45,46^). We then used a broken stick model and scree plots^47^ to determine how many PCs per species explained more variation in otolith shape than would be expected by chance alone.

**Figure 3:**
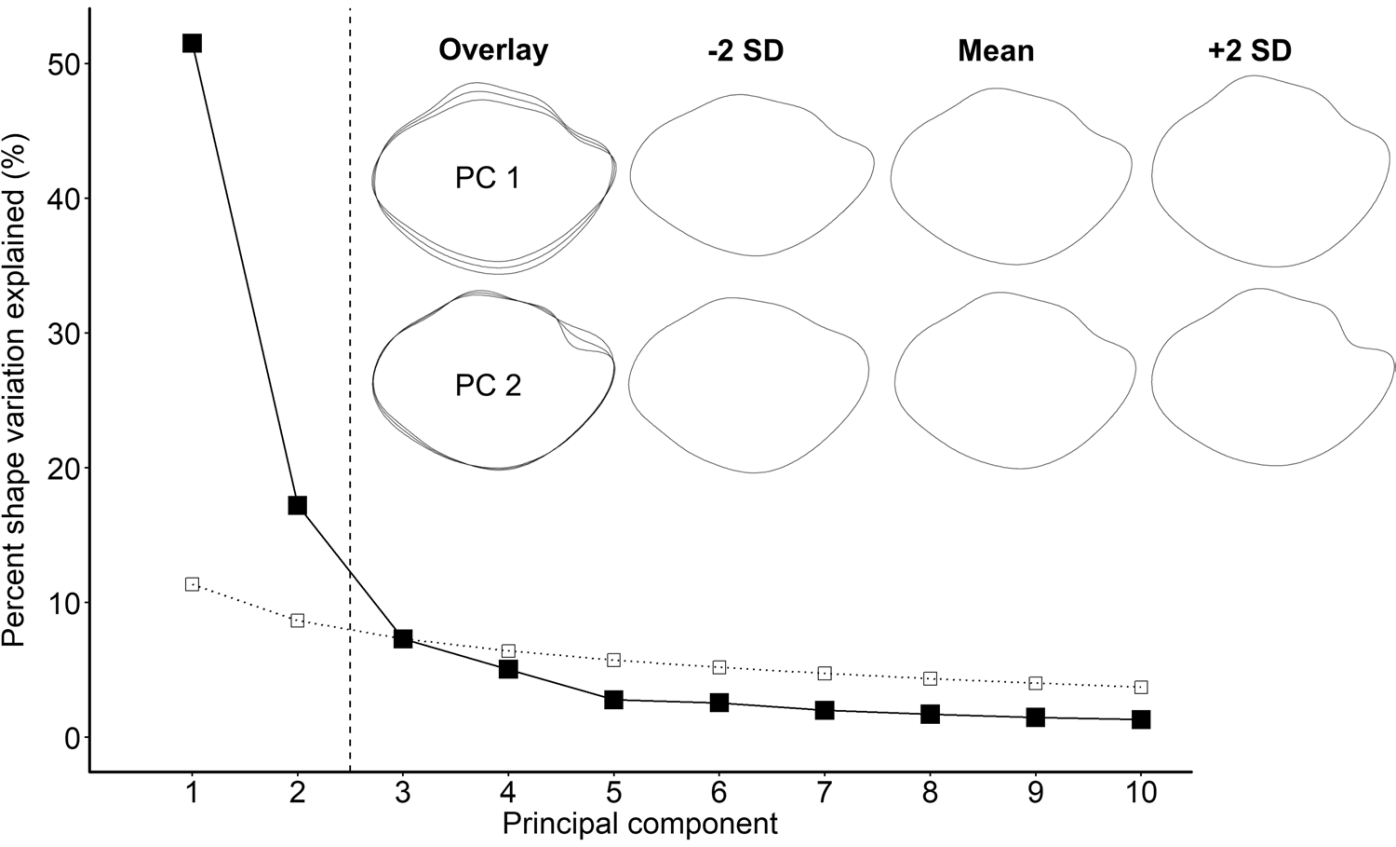
Scree plot illustrating how many principal components (filled squares) describe more variation in *Variabilichromis moorii* saccular otolith shape than by chance alone (i.e., the broken stick model, unfilled squares). The vertical dashed line represents the crossover between the data and the broken stick model. Otolith contour reconstructions visually illustrate the shape variation captured by each PC. Contours under the Mean column represent the average otolith shape for *V. moorii*. Contours on either side of the mean column illustrate the effect that increasing or decreasing each PC by two standard deviations has on otolith shape. Contours under the Overlay column allow for easier visualization of the shape variation captured by each PC.

For *V. moorii*, the first two PCs explained more variation than expected by chance (Fig. 3). For *N. caudopunctatus*, *N. savoryi*, and *N. pulcher* the first three PCs explained significantly more variation in shape than expected by chance. However, we focused on analyzing only the first two PCs for each species. Doing so allowed us to include all species in our analyses, without having to sometimes omit *V. moorii*. In addition, the first two PCs accounted for a large amount of shape variation (*N. caudopunctatus*, PC1: 43.5%, PC2: 14.4%; *N. pulcher*, PC1: 43.0%, PC2: 19.7%; *N. savoryi*, PC1: 41.4%, PC2: 17.9%; *V. moorii*, PC1: 51.5%, PC2: 17.2%), and these could easily be visually distinguished from one another. In all species investigated, PC1 and PC2 described similar axes of shape variation; PC1 described variation in otolith height (along the dorsal–ventral axis, see Fig. 3 and Supplementary Figs. S1–S3), while PC2 described variation in how pronounced the anterior notch was (see Fig. 3 and Supplementary Figs. S1–S3).

### Testing for sex differences across field sites

Otolith shape is known to differ between the sexes in certain species (e.g. silver hake *Merluccius bilinearis*^48^; plainfin midshipman, *Porichthys notatus*^8^), and so we first tested for sex differences in otolith shape (PC1 and PC2 scores) and did this for each field site and each species separately. We fit several linear mixed effects models, including PC as the response variable. Otolith surface area (µm^2^, scaled) and sex were included as fixed predictor variables and their interaction was tested for, though we dropped it from the models if non-significant. Because most fish contributed more than one otolith to our dataset, we included fish ID in the models as a random intercept. This process resulted in 24 separate tests for sex differences (two PCs, four species, three field sites), and so we implemented a conservative Bonferroni correction to maintain a family-wise error rate of 0.05. Therefore, we set our α threshold to 0.002 here.

Supplementary Figs. S4–S7 illustrate sex differences broken down by field site and species. *N. caudopunctatus* females had higher PC1 scores than males at Mutondwe Island (intercept: Est. ± SE = 0.036 ± 0.006, *t*_45.8_ = 6.0, *P* < 0.0001). *N. savoryi* females had higher PC1 scores than males at Mutondwe Island (intercept: Est. ± SE = 0.028 ± 0.005, *t*_48.6_ = 5.2, *P* < 0.0001). *N. pulcher* females had higher PC1 scores than males at Katukula (intercept: Est. ± SE = 0.036 ± 0.006, *t*_*42.0*_ = 6.2, *P* < 0.0001). No other contrasts were significant at the α = 0.002 significance threshold. Because of the strong sex differences that we found for certain species at certain field sites, we opted to analyze each sex separately in the coming analyses.

### Do fish species show consistent site differences in saccular otolith shape?

We tested whether, across species, saccular otolith shape showed consistent differences between field sites. That is, we asked whether otoliths sampled from certain sites had higher or lower PC scores compared to otoliths sampled from other sites, and whether these differences were consistently expressed across species. To do this, we fit a linear mixed effects model, for each sex separately, and included PC (either PC1 or PC2) as the response variable. Otolith surface area (µm^2^, scaled within each species), species, and field site were included as fixed predictor variables and fish ID was again included as a random intercept. We included the interaction term between species and field site in the model and then tested for pairwise marginal mean differences between each field site within each species (using the ‘emmeans’ R package^49^).

### Does saccular otolith shape variation covary with neutral genetic differentiation between populations?

Whole genomic DNA was extracted from the caudal fin clips taken in the field following a rapid Chelex protocol^50^, and the most variable part of the mitochondrial control region was amplified and sequenced according to the protocols described in^24^ and^19^. Sample sizes per population ranged from 24 to 33 (mean N = 29.6, Supplementary Table S1) and alignments comprised 357 bp (*N. caudopunctatus*), 392 bp (*N. savoryi*), 407 bp (*V. moorii*) and 409 bp (*N. pulcher*). Our use of the mitochondrial control region as a marker to estimate neutral population differentiation was guided by the following observations. First, mitochondrial sequence diversity in vertebrates is generally in accordance with expectations for neutral evolution^51,52^. More specifically, previous studies of Lake Tanganyika cichlids from the same geographic regions as investigated here, which used the same part of the mitochondrial control region, found a good agreement between estimates of neutral pairwise population differentiation and expectations based on paleohydrogeological data, habitat structure and degrees of stenotopy observed in the different species^20,21,24^. Since habitat barriers and lake level fluctuations are unlikely to have a sex-specific effect on gene flow between the sites investigated in this study, we expect the maternally inherited mitochondrial markers to reflect overall population structure. This is supported by agreement between mitochondrial and nuclear marker-based reconstructions of population structure in cichlid species within the same geographic area, including in *V. moorii*, one of our focal species in this study^19,24,29^.

We used Arlequin 3.5.1.2^53^ to calculate estimates of pairwise population differentiation based on uncorrected genetic distances between haplotypes (Φ_ST_^54^). Statistical significance was evaluated based on 1,023 permutations. We used PopART^55^ to draw haplotype networks based on statistical parsimony^56^ (Supplementary Figs. S8–S11). We chose Φ_ST_ over alternative estimators, because it integrates sequence divergence into an analysis of molecular variance and is not biased by genetic diversity^57^. In one population of *N. savoryi* (Kalambo), three haplotypes (four samples) were very divergent from the remaining network (see Supplementary Fig. S10) and most likely represent introgression from another Lamprologini species^58^. These putatively introgressed haplotypes were not included in the Φ_ST_ calculations, as they would inflate the estimate of within-population sequence divergence. Sequences are deposited in GenBank under accession numbers MT551233—MT551591.

We then tested whether the shape *difference* in saccular otoliths between *pairs* of populations varied according to the neutral *genetic differentiation* between the same population pairs. In particular, we expected that if otolith shape were genetically determined but selectively neutral, then there would be stronger shape differences between populations as the neutral genetic differentiation between those populations increased. Because otolith shape changes across ontogeny (i.e. changes as otoliths grow), we regressed otolith shape against otolith size and then extracted two ‘difference measures’: (1) *intercept* differences and (2) *slope* differences between each pair of populations (i.e. Mutondwe Island vs. Kalambo, Mutondwe Island vs. Katukula, and Kalambo vs. Katukula, see below for details).

To obtain intercept and slope differences between each population pair, we fit a series of linear mixed effects models. We ran separate models for each sex, species, and PC (two sexes, four species, two PCs = 16 models). We included PC (scaled) as the response variable, and otolith surface area (µm^2^, scaled) and field site as fixed predictor variables. Fish ID was included as a random intercept. We ran all of these models twice, once allowing for the interaction between otolith surface area and field site (to extract estimates of slope differences), and again omitting the interaction (this time to extract estimates of intercept differences).

We therefore obtained four ‘difference measures’ for each sex (eight in total) that described how divergent otolith shape was between each pair of populations: (1) difference in PC1 intercepts, (2) difference in PC2 intercepts, (3) differences in PC1 slopes, and (4) difference in PC2 slopes. To test whether these difference measures varied with the neutral genetic differentiation between populations, we fit a multivariate Bayesian linear mixed effects model (using the ‘blme’ R package^59^). All eight difference measures were fit as response variables (scaled), with neutral genetic differentiation, Φ_ST_, as a continuous predictor variable. We included two nested random effects: (1) allowing intercepts to vary within species and among difference measures within species, and (2) allowing intercepts to vary within population pairs and among difference measures within population pairs. Significance was estimated by calculating 95% confidence intervals for the effect size of genetic differentiation for each difference measure and then assessing whether any of them excluded zero. These confidence intervals were visualized using a coefficient plot that was built using the ‘dotwhisker’ R package^60^.

## Results

### Fish species show consistent site differences in saccular otolith shape

**Figure 4:**
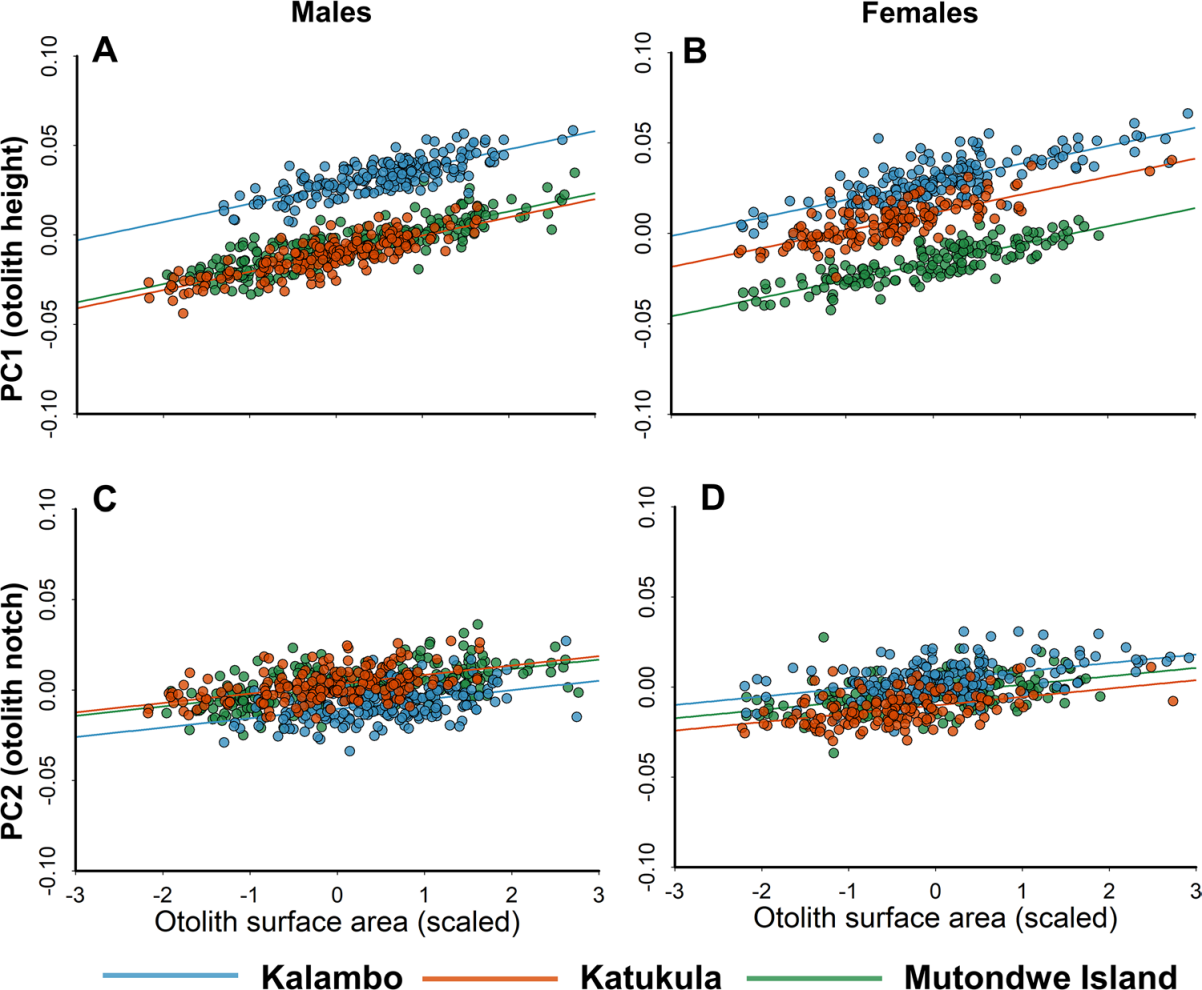
Site differences in saccular otolith shape as described by principal components (PC) 1 and 2. (**A**,**C**) Show male shape data, while (**B**,**D**) show female shape data. Plots show partial residuals from linear mixed-effects models (generated using ‘visreg’ package in R^79^). Note that otolith size was scaled (mean = 0, std. dev. = 1) within each species for these analyses.

Saccular otolith height (as captured by PC1) and the prominence of the anterior notch (as captured by PC2) increased with increasing otolith size in both males and females across species (Table 2; Supplementary Table S2). Saccular otolith height (PC1) differed between sites highly consistently: often in the same direction, for all species, and in both sexes. However, this pattern was far less consistent for the otolith anterior notch (PC2) (Fig. 4; Supplementary Table S2). See Supplementary Figs. S12 (for PC1) and S13 (for PC2) for partial residuals plots of these data split by species.

**Table 2:** Site comparisons.

Parameter					Estimate	Standard error	t-value, df	*P*
**Model: Males, PC1 (otolith height)**								
Otolith surface area (scaled within species)					0.010	0.0014	7.32, 340.6	**< 0.0001**
Species: *Neolamprologus caudopunctatus*								
Mutondwe vs. Kalambo					− 0.050	0.0060	− 8.31, 320	**< 0.0001**
Mutondwe vs. Katukula					− 0.050	0.0064	− 7.90, 318	**< 0.0001**
Kalambo vs. Katukula					− 0.00055	0.0061	− 0.091, 319	0.99
Species: *Neolamprologus pulcher*								
Mutondwe vs. Kalambo					− 0.046	0.0062	− 7.31, 315	**< 0.0001**
Mutondwe vs. Katukula					− 0.0073	0.0064	− 1.13, 320	0.49
Kalambo vs. Katukula					0.038	0.0060	6.38, 321	**< 0.0001**
Species: *Neolamprologus savory*								
Mutondwe vs. Kalambo					− 0.021	0.0062	− 3.41, 327	**0.0021**
Mutondwe vs. Katukula					− 0.044	0.0055	− 7.96, 330	**< 0.0001**
Kalambo vs. Katukula					− 0.023	0.0060	− 3.80, 326	**0.0005**
Species: *Variabilichromis moorii*								
Mutondwe vs. Kalambo					− 0.035	0.0056	− 6.23, 317	**< 0.0001**
Mutondwe vs. Katukula					0.0033	0.0055	0.60, 320	0.82
Kalambo vs. Katukula					0.038	0.0064	5.95, 320	**< 0.0001**
Random effects:	Group		Variance		Standard deviation			
	Fish ID		0.0004227		0.02056			
	Residual		0.00008038		0.008966			
**Model: Females, PC1 (otolith height)**								
Otolith surface area (scaled within species)					0.010	0.0016	6.18, 266.6	**< 0.0001**
Species: *Neolamprologus caudopunctatus*								
Mutondwe vs. Kalambo					− 0.034	0.0069	− 4.86, 254	**< 0.0001**
Mutondwe vs. Katukula					− 0.026	0.0067	− 3.85, 259	**0.0004**
Kalambo vs. Katukula					0.0078	0.0071	1.10, 254	0.51
Species: *Neolamprologus pulcher*								
Mutondwe vs. Kalambo					− 0.044	0.0062	− 7.16, 252	**< 0.0001**
Mutondwe vs. Katukula					− 0.027	0.0068	− 4.04, 250	**0.0002**
Kalambo vs. Katukula					0.017	0.0067	2.56, 251	**0.0297**
Species: *Neolamprologus savoryi*								
Mutondwe vs. Kalambo					− 0.010	0.0068	− 1.50, 255	0.29
Mutondwe vs. Katukula					− 0.027	0.0071	− 3.74, 254	**0.0007**
Kalambo vs. Katukula					− 0.017	0.0073	− 2.25, 249	0.065
Species: *Variabilichromis moorii*								
Mutondwe vs. Kalambo					− 0.044	0.0069	− 6.30, 260	**< 0.0001**
Mutondwe vs. Katukula					0.013	0.0076	1.74, 264	0.19
Kalambo vs. Katukula					0.057	0.0071	8.02, 266	**< 0.0001**
Random effects:		Group		Variance	Standard deviation			
		Fish ID		0.0004623	0.02150			
		Residual		0.00009917	0.009958			

Results of marginal means contrasts from linear mixed effects models comparing saccular otolith shape (as captured by PC1) between populations (Kalambo, Mutondwe, and Katukula) for each species, while controlling for otolith size (measured by surface area). Male and female models were run separately due to strong sex differences (see “Methods”). Significant results at α = 0.05 are shown in bold. Multiple comparisons are accounted for using the Tukey method.

### Saccular otolith shape variation does not covary with neutral genetic differentiation between populations

**Figure 5:**
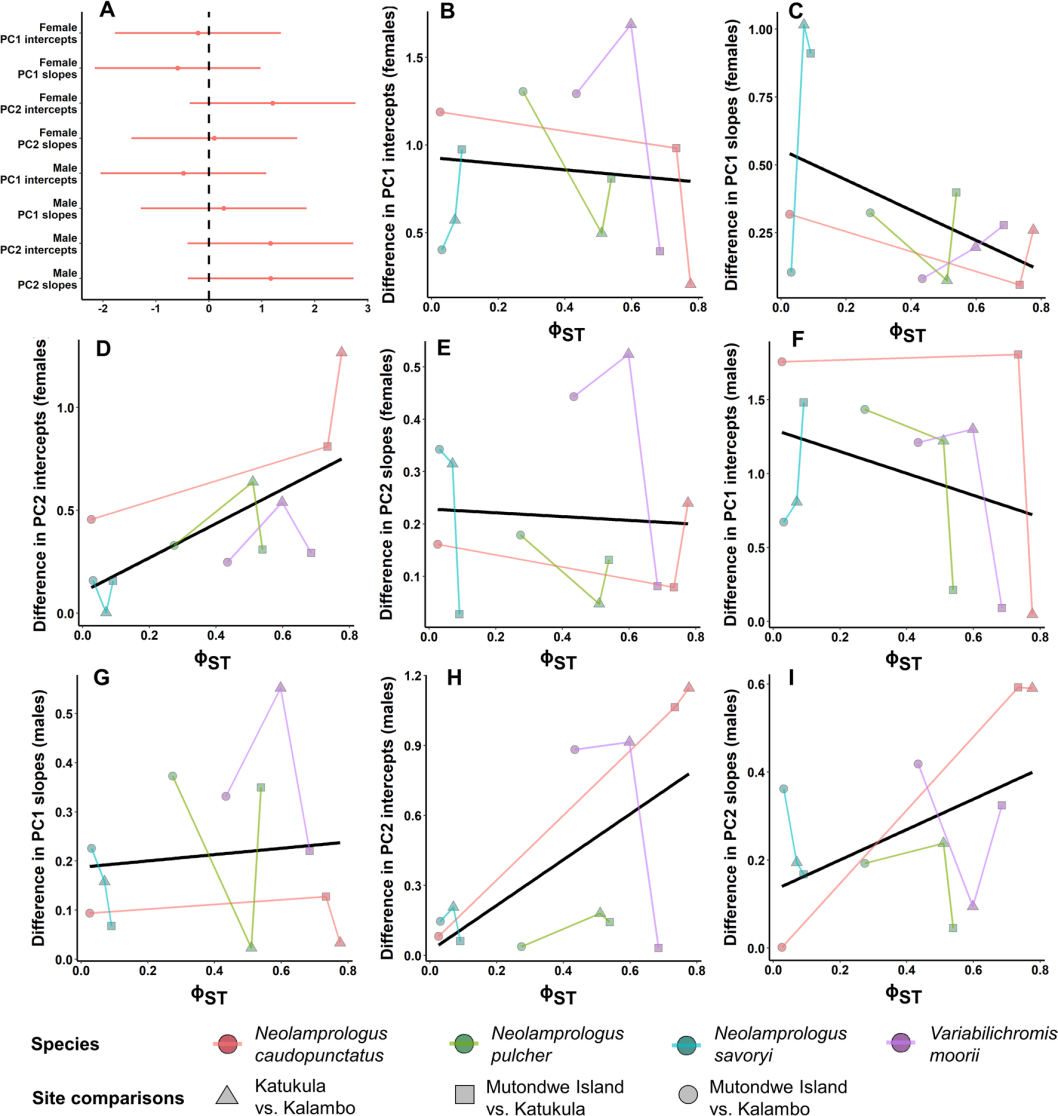
Comparisons of saccular otolith shape versus neutral genetic variation. (**A**) 95% confidence intervals calculated for the effect size of the neutral genetic differentiation between populations (Φ_ST_) on each saccular otolith shape ‘difference measure’ (see “Methods” for details). (**B**–**I**) Linear mixed effects model regression fits for each otolith shape difference measure, to help visualize the relationship between population genetic differentiation and otolith shape.

Population pairwise Φ_ST_ values ranged from 0.026 to 0.776 for *N. caudopunctatus*, 0.274–0.539 for *N. pulcher*, 0.031–0.091 for *N. savoryi*, and 0.434–0.685 for *V. moorii* and were significantly different from zero except for the comparison between Kalambo and Mutondwe in *N. caudopunctatus* (Fig. 5, see Supplementary Table S1 for pairwise population Φ_ST_ values). Population differences in saccular otolith shape did not correlate significantly with neutral genetic differentiation, as quantified with Φ_ST_; none of the 95% confidence intervals for the effect size of Φ_ST_ calculated for any of the eight otolith shape ‘difference measures’ excluded zero (Fig. 5A). While none of the difference measures showed statistically significant slopes across the range of population Φ_ST_ values (Fig. 5B–I), one difference measure tended negatively (Fig. 5C) and three measures tended positively (Fig. 5D,H,I), corroborated by a visual inspection of the coefficient plot (Fig. 5A).

## Discussion

Saccular otolith shape variation is often assumed to be adapted to the local environment. This has often been studied via an ecomorphology approach, in which otolith morphology is compared across field sites and studied in relation to different environmental factors. However, these studies often use either a single focal species sampled from multiple sites, or multiple species each typically found in and sampled from separate, non-overlapping sites or habitat types; in both cases, an axis of replication is lacking. A more powerful approach, as we have done here, is to sample multiple sympatric species from multiple sites, and test for consistent between-site variation in otolith shape. Furthermore, many studies lack a direct measure of neutral genetic differentiation between their populations, precluding any formal test for whether saccular otolith shape variation is consistent with random genetic drift. Our chosen set of species and populations are therefore suitable for studying whether otolith shape responds to environmental variation or represents neutral morphological variation.

We detected clear site-differences in saccular otolith shape and many of these differences were qualitatively consistent across species. For example, otoliths from our Kalambo field site were consistently and significantly taller than otoliths from Mutondwe Island for all species and both sexes (with the exception of *N. savoryi* females). Our four study species were sampled from populations that live in rocky littoral habitats. Deviations in any number of a wide array of environmental variables could, in principle, underlie our site differences in otolith shape; within-sites, our species should all have experienced similar depths, temperatures, food availability, and water chemistry profiles (all variables that have been previously shown to be associated with otolith shape variation, e.g.^13,15,61–66^), but these variables may have differed subtly between sites. Our results support an ecomorphological perspective, suggesting that the local ecological conditions give rise to particular otolith shapes^3^. We cannot, however, disentangle whether our site-differences in saccular otolith shape are driven by genetic evolution or phenotypic plasticity; a common-garden experiment in which offspring from different populations are raised together under a standard environment would be needed to make this distinction. Overall, our results indicate that multi-species communities of fish can possess unifying similarities in their saccular otolith shapes due to geographic overlap. If the local soundscape imposes selection on the form and function of the fish auditory system, then it could be expected that sympatric species, that occupy similar niche space and make similar use of their surrounding soundscape, will express phenotypic similarities in auditory structures, including otoliths.

Site differences were primarily detected in saccular otolith height (i.e. PC1), and not in the anterior notch (PC2), suggesting that otolith height may be, in-part, locally attuned to the soundscapes at our field sites, while the anterior notch is not, or is to a lesser degree. There is increasing evidence from mathematical models^18^ and experimental work^67,68^ that the shape of otoliths influences their patterns of movement relative to the sensory epithelium within the inner ear end organs, with likely consequences for the auditory and vestibular systems. How exactly otolith height and notching respectively affect otolith motion (along with other common axes of otolith shape variation) would be a fruitful avenue for future research.

Differences between our populations in saccular otolith shape did not correlate significantly with their level of neutral genetic differentiation. We had predicted that if saccular otolith shape variation were heritable and selectively neutral, then we would detect a positive correlation between our shape ‘difference measures’ and Φ_ST_. While it can be difficult to argue for any inference based solely on the absence of a statistically significant relationship, our shape versus Φ_ST_ comparisons are consistent with our other results, which show strong site-differences in shape across species suggestive of locally-adapted saccular otolith forms. In fact, we found the weakest evidence for consistent site differences in the anterior notch (i.e. PC2), which also displayed notable positive trends between three of its four shape difference measures and Φ_ST_ (Fig. 5D,H,I). Taken together, this suggests that saccular otolith height requires more fine-tuning to environmental conditions than does the anterior notch. A biological significance for saccular otolith shape has previously been alluded to by studies showing that shape differences between populations can increase in magnitude with increasing geographic separation^69–73^, though it is rare for the relationship between otolith shape and neutral genetic variation to be directly considered. In our study, the lack of any statistically clear correlations between our otolith shape ‘difference measures’ and neutral genetic differentiation (i.e. mitochondrial control region), implies that factors, such as adaptation to local acoustic environments, play a larger role than random drift in determining otolith shape.

Patterns of genetic differentiation estimated from a mitochondrial sequence marker may fail to reflect the evolutionary history of populations, if mitochondrial genomes evolve under the influence of selection or sex-biased gene flow, or experience very idiosyncratic drift^74^. For meaningful comparisons between otolith shape differences and genetic differentiation estimates in our study, it is important that the variation in Φ_ST_ values between pairs of populations reflects the variable degrees of differentiation between those population pairs (i.e., Φ_ST_ values should be relatively lower between populations that diverged recently or are connected by higher rates of gene flow, and Φ_ST_ values should be relatively higher between populations that have been strictly isolated for longer). The fact that the differentiation estimates in our analysis showed the expected patterns (based on habitat structure and paleohydrological conditions; see “Methods”, *Selection of field sites and study species*) suggests that this was achieved by our sequence dataset. As predicted, pairwise Φ_ST_ values in all four species were highest in comparisons across Mbete Bay, which is a stronger and more persistent barrier to gene flow for rock-dwelling cichlids than Chituta Bay^24,25^. Furthermore, species differences in the estimated differentiation levels are consistent with species-specific traits related to the evolution of neutral population structure (as summarized in Konings^36^). For instance, in the strongly stenotopic rock-dwelling *V. moorii*, the Φ_ST_ value between Mutondwe and Kalambo was almost as high as the Φ_ST_ values across Mbete Bay, whereas we detected no significant differentiation between Mutondwe and Kalambo in the ecologically more versatile *N. caudopunctatus*. In *N. pulcher*, differences in the gill-cover markings between populations imply restricted gene flow, as confirmed by the high Φ_ST_ values. Finally, *N. savoryi* expand into greater depths than the other species, which may facilitate connectivity among populations irrespective of shoreline heterogeneity and explain their low differentiation and high genetic diversity (Supplementary Fig. S10).

We found strong sex differences in saccular otolith shape for certain species at particular sites. Although the sex differences were not necessarily limited to one species or one population, they were mostly detected for otolith height (PC1). Interestingly, when sex differences were detected, females possessed taller saccular otoliths than males (Supplementary Figs. S4–S7). Saccular otolith height was also the aspect of shape that differed most strongly between sites, supporting the idea that otolith height, more so than the anterior notch, imparts a functional consequence for the fish auditory (and perhaps vestibular) system. It is currently unclear why such pronounced site-specific sex differences might exist. When sex differences in otolith shape have been uncovered in the past (e.g. Atlantic cod, *G. morhua*^7,75^; flatfish spp.^9^; plainfin midshipman, *P. notatus*^8^; *Neobythites gilli*^76^), a common explanation is that the sexes likely differ with respect to their somatic growth rates and metabolic rates. Growth rates, energy storage, and metabolism have rarely been explicitly compared between the sexes in our study species, but when they have, males and females have scored similarly (e.g. *N. pulcher*^77^). An interesting avenue for future research will be to investigate whether vocal communication occurs in our species (sound production by *N. pulcher* has been documented^40^), whether the vocal and/or auditory capabilities differ between the sexes, and how any such sex effects are associated with further (neuro)anatomical differences. Our observations show that sex differences in saccular otolith shape can be found on a seemingly case-by-case basis. We therefore emphasize the importance of accounting for sex whenever possible, and we highlight the dangers of sampling from a single population and then generalizing conclusions to other populations or to the whole species.

In summary, we found that multiple fish species can display similarities in saccular otolith shape dependent on the field site that they were collected from. Furthermore, the substantial shape differences that we found did not correlate significantly with our measures of neutral genetic differentiation between the populations. In particular, we point to the apparent importance of otolith height, as this was the aspect of saccular otolith shape that differed most dramatically between sites, showed the weakest correlation to neutral genetic differentiation, and also showed prominent sex differences. Our data are therefore consistent with the ecomorphology hypothesis for saccular otolith shape, implying that otolith shape constitutes non-neutral variation in response to local environments.

## Supplementary information

Supplementary Information 1.

## Data Availability

All analyses in this paper can be reproduced using the data uploaded to Dryad (https://doi.org/10.5061/dryad.mgqnk98wz).
