# Supplementary material for: Congruent geographic variation in saccular otolith shape across multiple species of African cichlids
Source: Sci Rep. 2020 Jul 30;10:12820. doi: 10.1038/s41598-020-69701-9 (PMC7393159; doi:10.1038/s41598-020-69701-9)
Supplement: Supplementary file 1 — Supplementary Information 1. [file 41598_2020_69701_MOESM1_ESM.pdf]

Congruent geographic variation in saccular otolith shape across multiple species of African cichlids

Aneesh P. H. Bose, Holger Zimmermann, Georg Winkler, Alexandra Kaufmann, Thomas Strohmeier, Stephan Koblmüller, Kristina M. Sefc

SUPPLEMENTARY TABLES

Supplementary Table S1: Mitochondrial control region (i.e. D-loop) haplotype composition compared between our three field sites and given for each species. *N* = sample size in number of individuals sequenced, *h* = number of different mitochondrial haplotypes found, *He* = genetic diversity, *Nucleotide diversity* = average pairwise distance between sequences / length of sequence in bp.

Field site	*N*	*h*	*He*	*Nucleotide diversity*	Average pairwise distance	Pairwise Φ_{ST} (below diagonal) *P*-values (above diagonal)		
Neolamprologus caudopunctatus						Katukula	Mutondwe	Kalambo
Katukula	30	9	0.862	0.006	2.228	-	0	0
Mutondwe	30	10	0.892	0.005	1.680	0.734	-	0.104
Kalambo	30	4	0.579	0.003	1.090	0.776	0.026	-
Neolamprologus pulcher								
Katukula	32	21	0.970	0.016	6.738	-	0	0
Mutondwe	30	23	0.975	0.019	7.901	0.539	-	0
Kalambo	33	10	0.850	0.009	3.742	0.510	0.274	-
Neolamprologus savoryi								
Katukula	32	18	0.927	0.025	9.565	-	0	0
Mutondwe	26	19	0.975	0.020	7.729	0.091	-	0.023
Kalambo	28	22	0.974	0.028	11.087	0.070	0.031	-
Variabilichromis moorii								
Katukula	30	14	0.933	0.011	4.646	-	0	0
Mutondwe	24	17	0.971	0.006	2.254	0.685	-	0
Kalambo	30	11	0.858	0.007	2.936	0.598	0.434	-

Supplementary Table S2: Site comparisons. Results of marginal means contrasts from linear mixed effects models comparing saccular otolith shape (as captured by PC2) between populations (Kalambo, Mutondwe, and Katukula) for each species, while controlling for otolith size (measured by surface area). Male and female models were run separately due to strong sex differences (see Methods). Significant results at α = 0.05 are shown in bold. Multiple comparisons are controlled for using the Tukey method.

Parameter	Estimate	Standard Error	t-value, df	*P*
Model: Males, PC2 (anterior notch)				
Otolith surface area (scaled within species)	0.0052	0.0011	4.81, 322.5	**<0.0001**
Species: *Neolamprologus caudopunctatus*				
Mutondwe vs. Kalambo	-0.0047	0.0046	-1.03, 315	0.56
Mutondwe vs. Katukula	0.012	0.0049	2.50, 310	**0.034**
Kalambo vs. Katukula	0.017	0.0046	3.65, 313	**0.0009**
Species: *Neolamprologus pulcher*				
Mutondwe vs. Kalambo	0.00056	0.0048	0.12, 306	0.99
Mutondwe vs. Katukula	0.0018	0.0050	0.36, 314	0.93
Kalambo vs. Katukula	0.0012	0.0047	0.27, 316	0.96
Species: *Neolamprologus savoryi*				
Mutondwe vs. Kalambo	0.0019	0.0048	0.40, 331	0.92
Mutondwe vs. Katukula	0.0028	0.0043	0.65, 336	0.79
Kalambo vs. Katukula	0.00091	0.0047	0.20, 328	0.98
Species: *Variabilichromis moorii*				
Mutondwe vs. Kalambo	0.012	0.0043	2.76, 308	**0.017**
Mutondwe vs. Katukula	-0.0019	0.0042	-0.45, 317	0.90
Kalambo vs. Katukula	-0.014	0.0049	-2.78, 316	**0.016**
Random effects:	Group	Variance	Standard Deviation	
	Fish ID	0.0002085	0.01444	
	Residual	0.0001196	0.01094	
Species: *Neolamprologus caudopunctatus*				
Mutondwe vs. Kalambo	-0.0049	0.0050	-0.97, 252	0.60
Mutondwe vs. Katukula	0.010	0.0049	2.06, 261	0.10
Kalambo vs. Katukula	0.015	0.0051	2.93, 251	**0.011**
Species: *Neolamprologus pulcher*				
Mutondwe vs. Kalambo	-0.0074	0.0045	-1.64, 248	0.23
Mutondwe vs. Katukula	0.0068	0.0049	1.39, 244	0.35
Kalambo vs. Katukula	0.014	0.0048	2.95, 245	**0.0097**
Species: *Neolamprologus savoryi*				
Mutondwe vs. Kalambo	0.0018	0.0049	0.36, 254	0.93
Mutondwe vs. Katukula	0.0058	0.0052	1.12, 250	0.50
Kalambo vs. Katukula	0.0040	0.0053	0.76, 241	0.73
Species: *Variabilichromis moorii*				
Mutondwe vs. Kalambo	0.0045	0.0051	0.90, 263	0.64
Mutondwe vs. Katukula	-0.0044	0.0056	-0.78, 271	0.71
Kalambo vs. Katukula	-0.0089	0.0052	-1.70, 276	0.21
Random effects:	Group	Variance	Standard Deviation	
	Fish ID	0.0002065	0.01437	
	Residual	0.0001188	0.01090	

SUPPLEMENTARY FIGURES

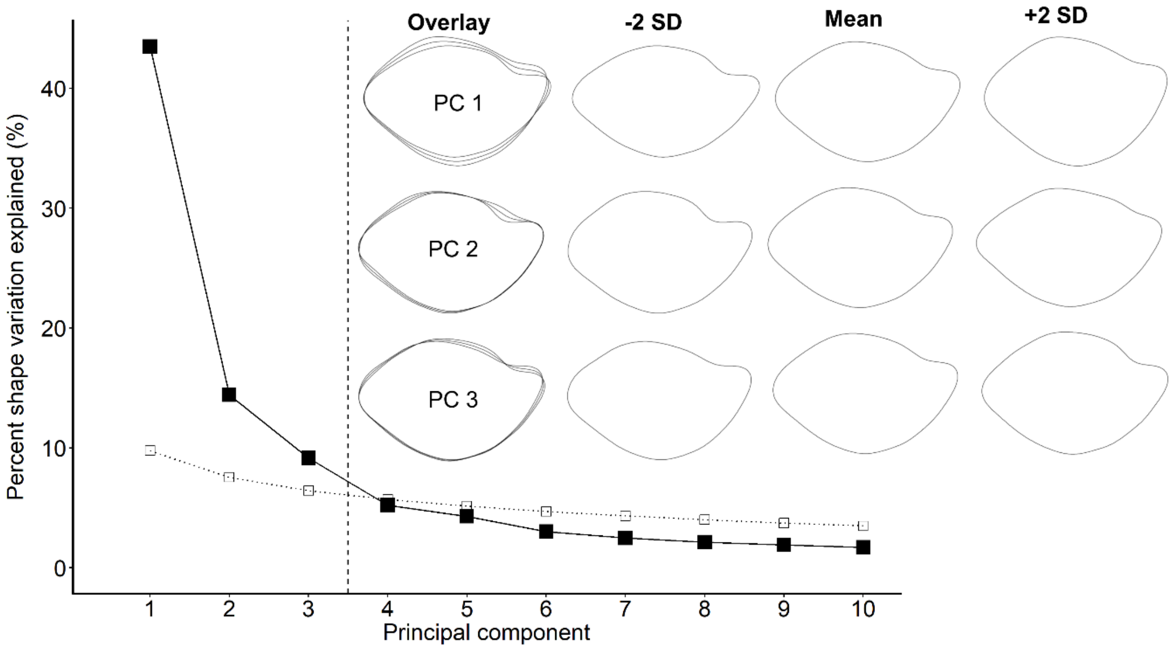

Supplementary Figure S1: Scree plot illustrating how many principal components (–■–) describe more variation in *Neolamprologus caudopunctatus* saccular otolith shape than by chance alone (i.e., the broken stick model, --□--). The vertical dashed line represents the crossover between the data and the broken stick model. Otolith contour reconstructions visually illustrate the shape variation captured by each PC. Contours under the Mean column represent the average otolith shape for *N. caudopunctatus*. Contours on either side of the mean column illustrate the effect that increasing or decreasing each PC by two standard deviations has on otolith shape. Contours under the Overlay column allow for easier visualization of the shape variation captured by each PC.

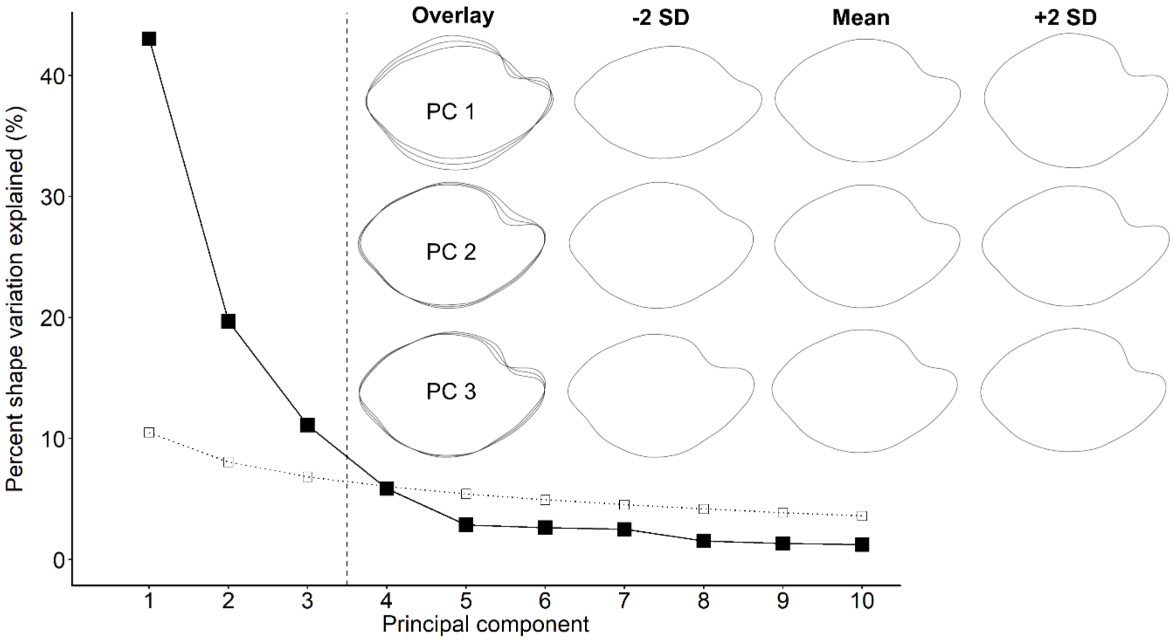

Supplementary Figure S2: Scree plot illustrating how many principal components (–■–) describe more variation in *Neolamprologus pulcher* saccular otolith shape than by chance alone (i.e., the broken stick model, --□--). The vertical dashed line represents the crossover between the data and the broken stick model. Otolith contour reconstructions visually illustrate the shape variation captured by each PC. Contours under the Mean column represent the average otolith shape for *N. pulcher*. Contours on either side of the mean column illustrate the effect that increasing or decreasing each PC by two standard deviations has on otolith shape. Contours under the Overlay column allow for easier visualization of the shape variation captured by each PC.

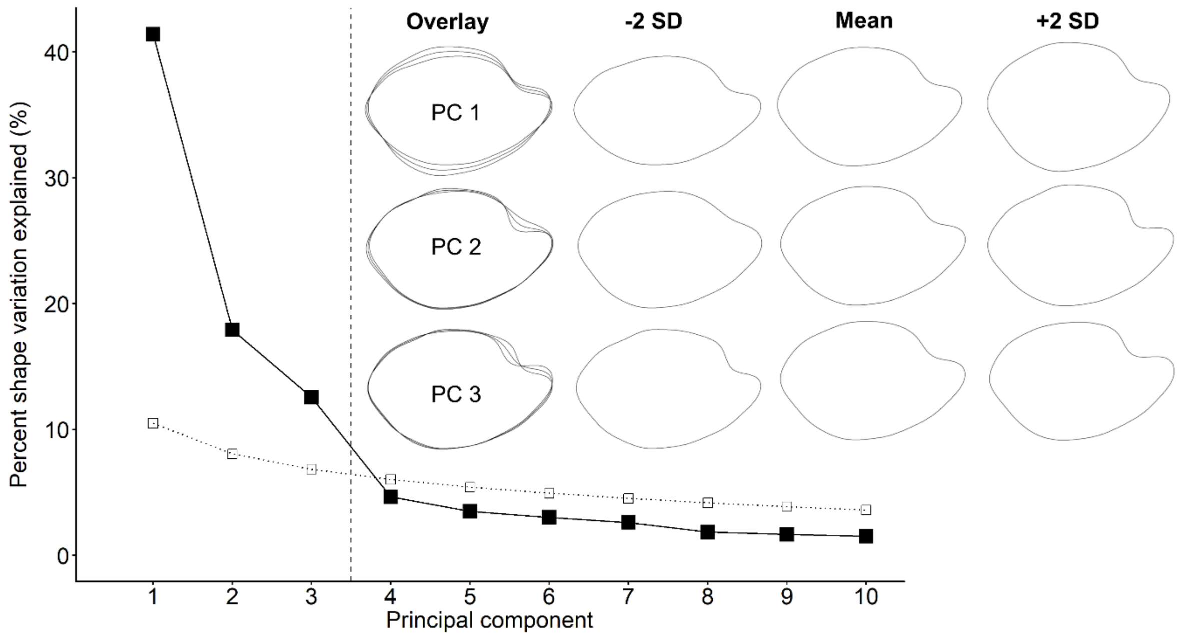

Supplementary Figure S3: Scree plot illustrating how many principal components (–■–) describe more variation in *Neolamprologus savoryi* saccular otolith shape than by chance alone (i.e., the broken stick model, --□--). The vertical dashed line represents the crossover between the data and the broken stick model. Otolith contour reconstructions visually illustrate the shape variation captured by each PC. Contours under the Mean column represent the average otolith shape for *N. savoryi*. Contours on either side of the mean column illustrate the effect that increasing or decreasing each PC by two standard deviations has on otolith shape. Contours under the Overlay column allow for easier visualization of the shape variation captured by each PC.

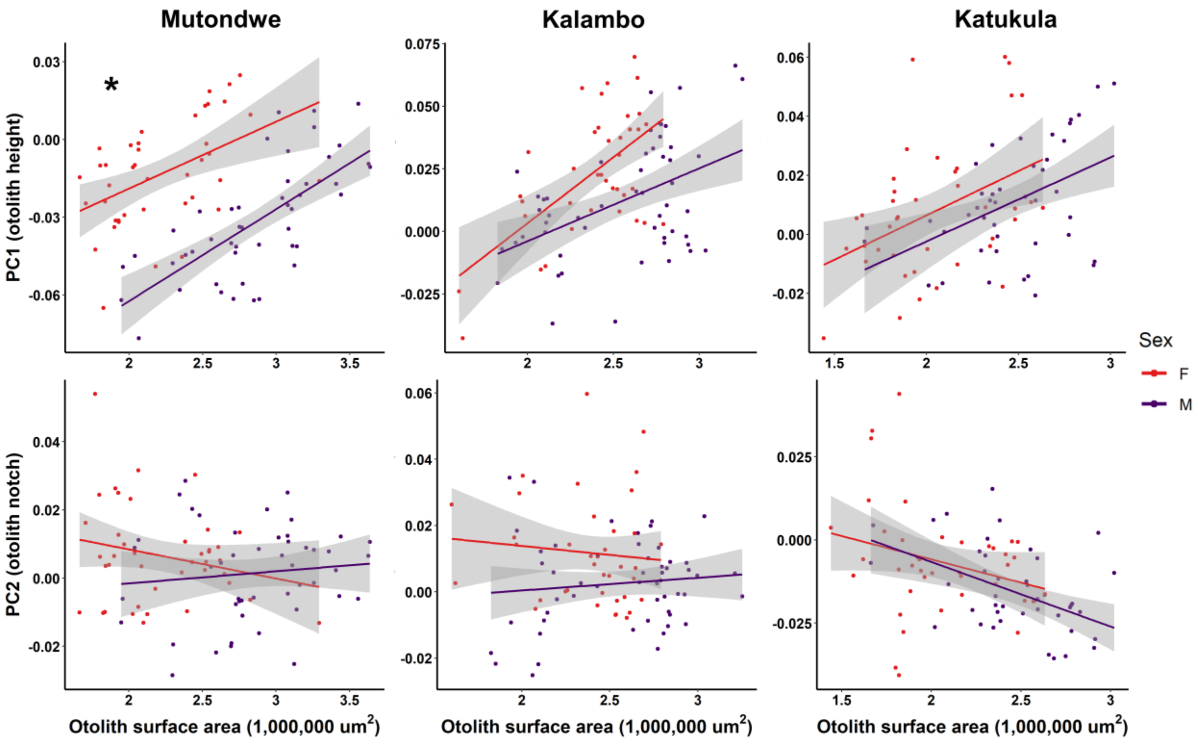

54 **Supplementary Figure S4: Sex effects.** Correlations between saccular otolith shape (as captured by two principal components, PC1 and PC2) and otolith size (surface area) for *Neolamprologus caudopunctatus*, broken down by field site and illustrating sex differences (indicated by *, see Methods).

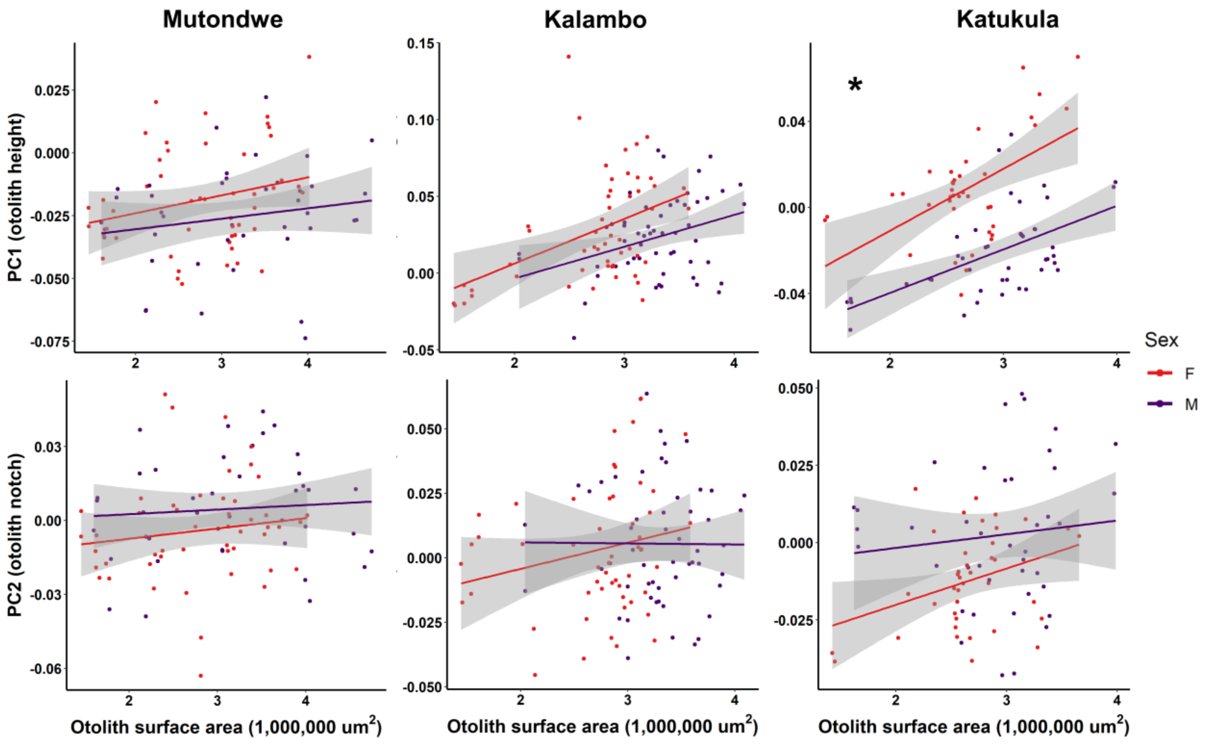

Supplementary Figure S5: Sex effects. Correlations between saccular otolith shape (as captured by two principal components, PC1 and PC2) and otolith size (surface area) for *Neolamprologus pulcher*, broken down by field site and illustrating sex differences (indicated by *, see Methods).

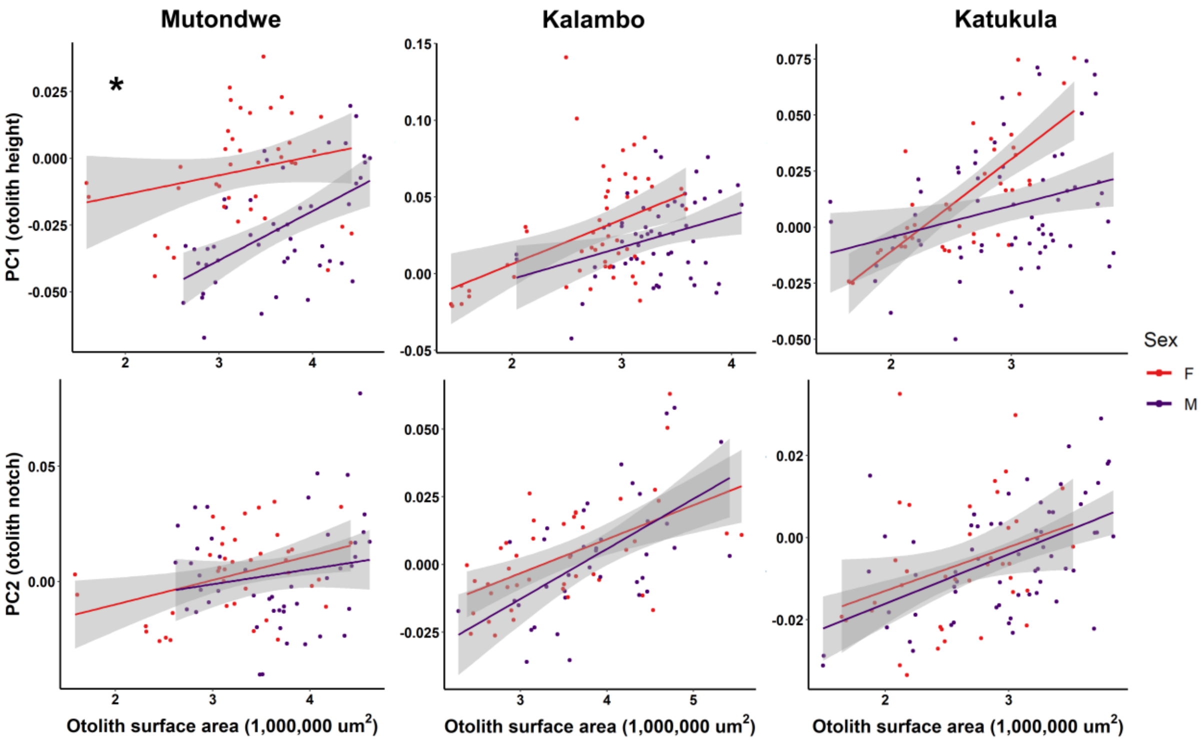

Supplementary Figure S6: Sex effects. Correlations between saccular otolith shape (as captured by two principal components, PC1 and PC2) and otolith size (surface area) for *Neolamprologus savoryi*, broken down by field site and illustrating sex differences (indicated by *, see Methods).

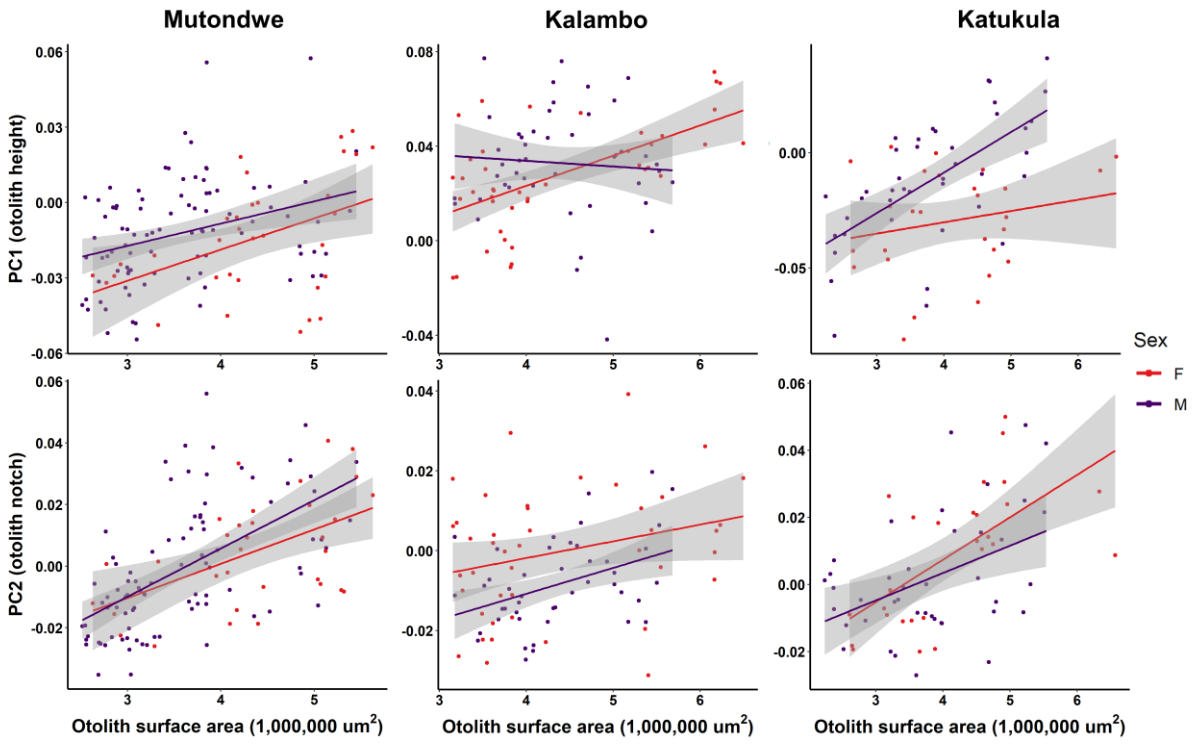

Supplementary Figure S7: Sex effects. Correlations between saccular otolith shape (as captured by two principal components, PC1 and PC2) and otolith size (surface area) for *Variabilichromis moorii*, broken down by field site and illustrating sex differences (indicated by *, see Methods).

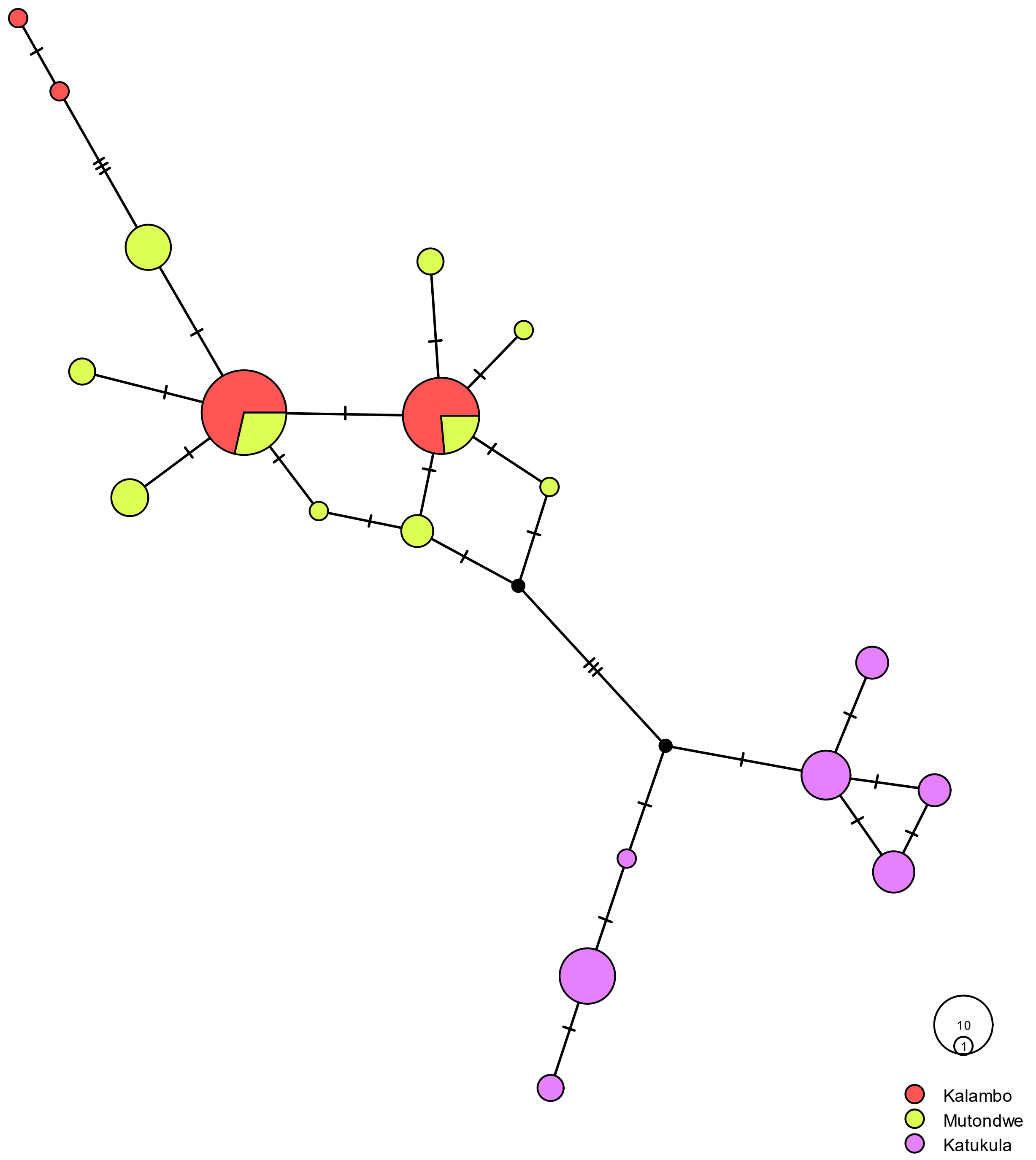

Supplementary Figure S8: Statistical parsimony network illustrating relationships among the *Neolamprologus caudopuncatus* control region haplotypes. Mutations are shown as hatch marks. Black dots indicate intermediate haplotypes not included in our dataset that are necessary to link all observed haplotypes to the network. Different colours correspond to the different field sites.

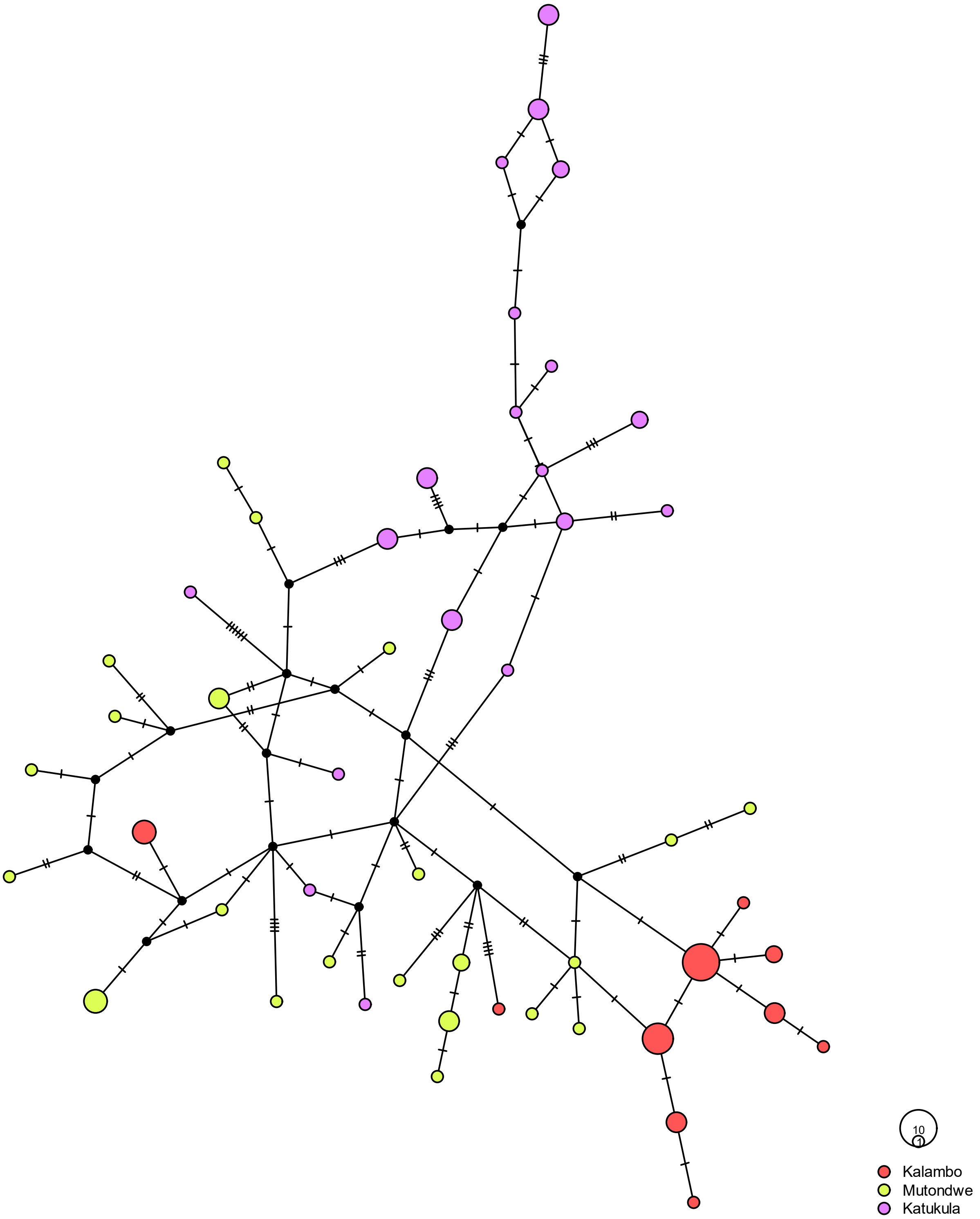

Supplementary Figure S9: Statistical parsimony network illustrating relationships among the *Neolamprologus pulcher* control region haplotypes. Mutations are shown as hatch marks. Black dots indicate intermediate haplotypes not included in our dataset that are necessary to link all observed haplotypes to the network. Different colours correspond to the different field sites.

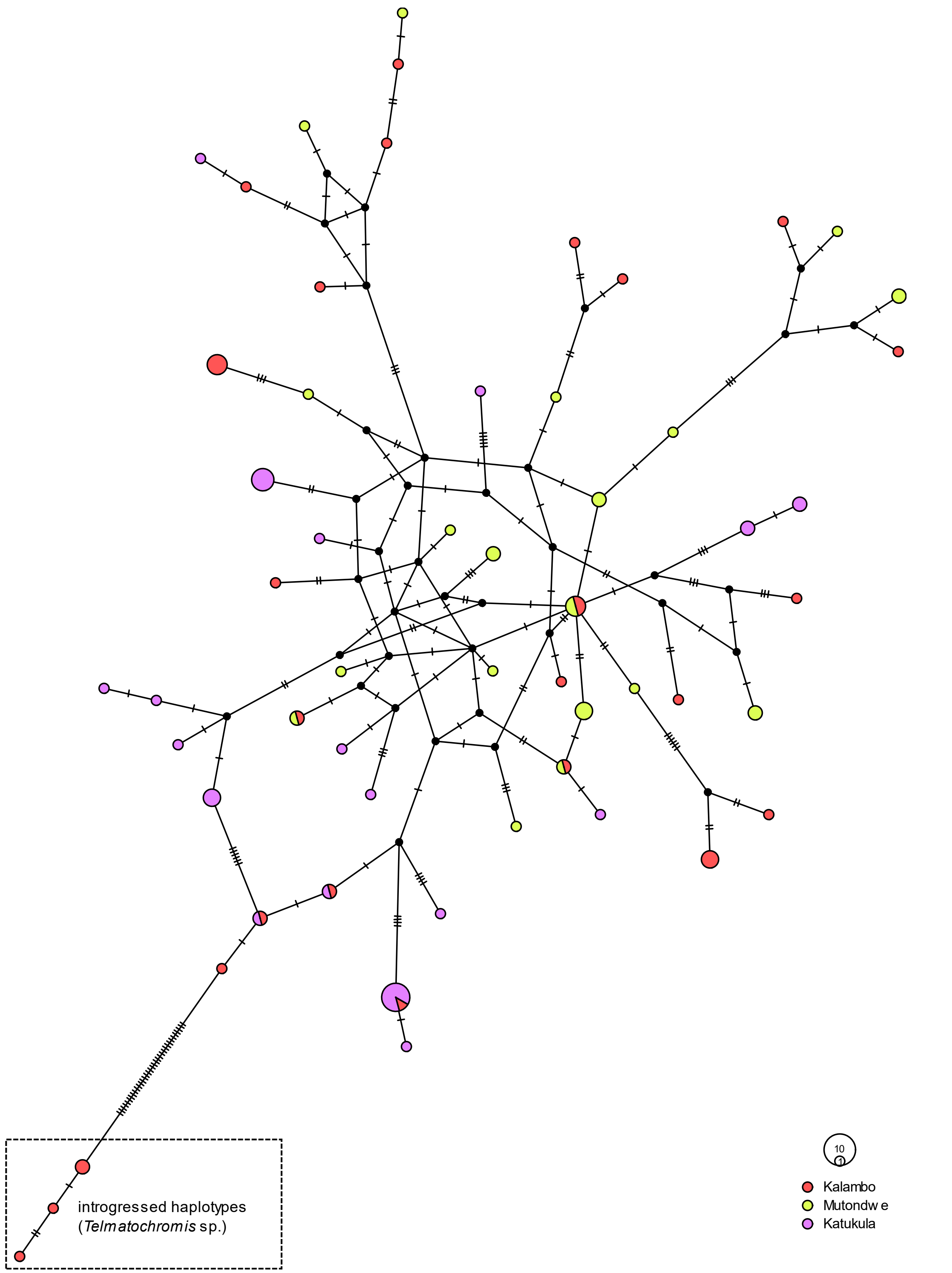

Supplementary Figure S10: Statistical parsimony network illustrating relationships among the *Neolamprologus savoryi* control region haplotypes. Mutations are shown as hatch marks. Black dots indicate intermediate haplotypes not included in our dataset that are necessary to link all observed haplotypes to the network. Different colours correspond to the different field sites. Four samples had introgressed haplotypes (from the *Telmatochromis brachygnathus*, *dhonti* & *temporalis* group).

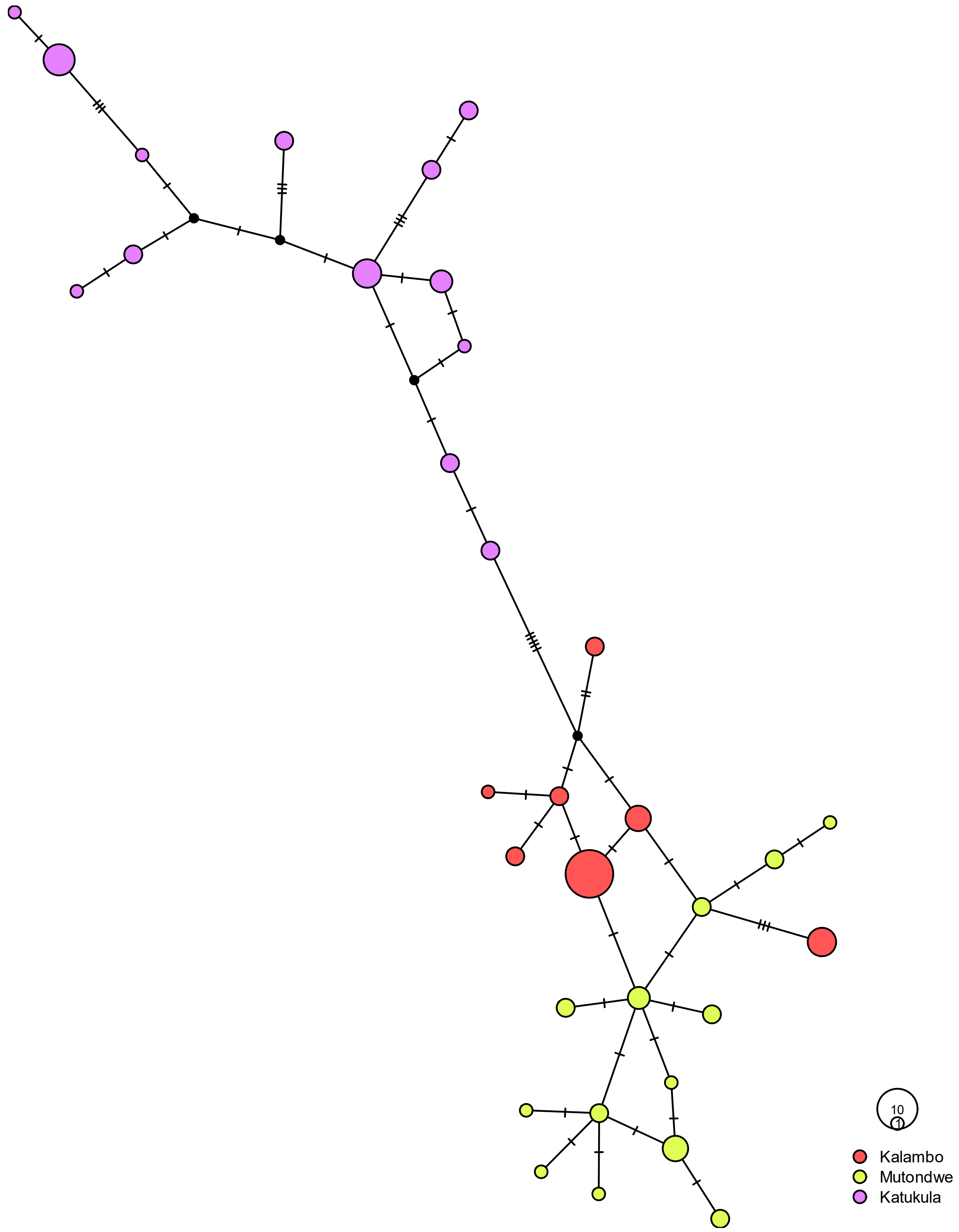

Supplementary Figure S11: Statistical parsimony network illustrating relationships of the *Variabilichromis moorii* control region haplotypes. Mutations are shown as hatch marks. Black dots indicate intermediate haplotypes not included in our dataset that are necessary to link all observed haplotypes to the network. Different colours correspond to the different field sites.

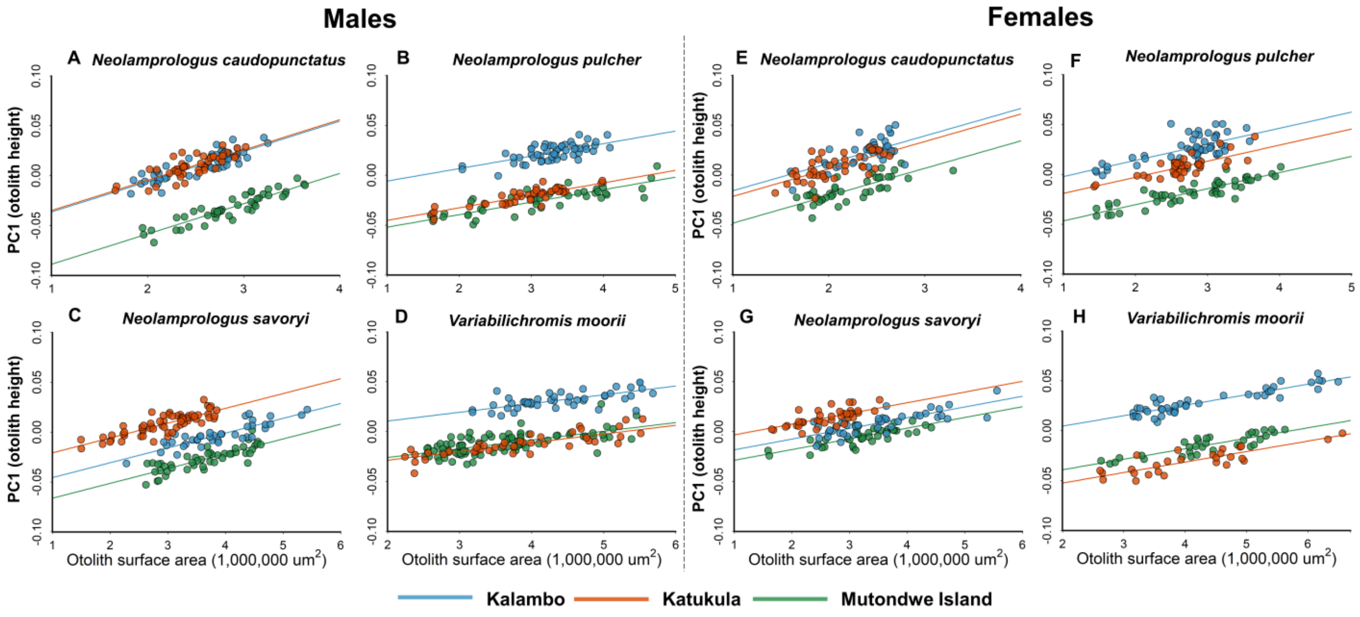

Supplementary Figure S12: Site differences in saccular otolith shape as described by principal component 1, broken down by species. Panels A – D show male shape data, while panels E – H show female shape data. Plots show partial residuals from linear mixed-effects models (generated using ‘visreg’ package in R, Breheny and Burchett 2017).

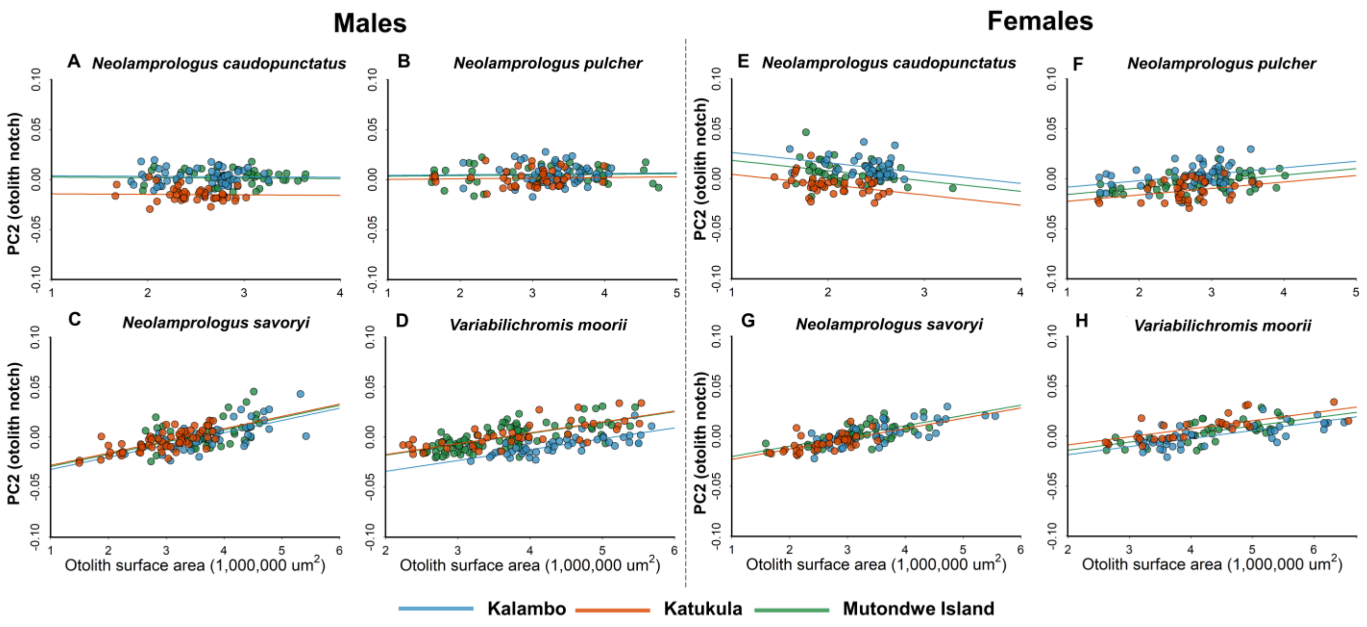

Supplementary Figure S13: Site differences in saccular otolith shape as described by principal component 2, broken down by species. Panels A – D show male shape data, while panels E – H show female shape data. Plots show partial residuals from linear mixed-effects models (generated using ‘visreg’ package in R, Breheny and Burchett 2017).

REFERENCES

Breheny, P. & Burchett, W. Visualization of regression models using visreg. *R J* **9**, 56-71 (2017).
